# The Power of Potentizing

**Published:** 1897-05

**Authors:** Malcolm Macfarlan

**Affiliations:** Philadelphia


### THE POWER OF POTENTIZING.

MALCOLM MACFARLAN, M. D., PHILADELPHIA.

It is believed by some that there is a growing tendency to doubt the curative action of medicines, as advised and prepared by Hahnemann during the later years of his life—remedies commonly called potencies by those who use them, and dilutions by those who refer to them.

If there is a positive healing power, peculiar to itself, evolved from a material substance, by shaking with water or other solvent, or by triturating with some constant harmless substance, like sugar of milk, then Hahnemann has demonstrated that there exists in matter so treated a curative force, developed by succussion and separation, which, so far as the limited reading or knowledge of the writer goes, Hahnemann was the first to practically demonstrate, and it can be proven in no other way than by comparatively high potencies.

The marvelous results of the subdivision of atoms, made by shaking with water, simply overwhelms one when a mathematical calculation is made, using the centesimal scale, and where at each change of fluid or potency the substance is divided by one hundred. Think of a grain of soluble material, divided so finely that the amount in the medicine used is represented by a fraction whose numerator is one and the denominator the same figure followed by a hundred, or even as now sometimes used, a thousand or more ciphers? The only correspondence to the inverted vastness of this is in the figures given by astronomers in which, for instance, it is said that it takes many years for light, traveling at the rate of about two hundred thousand miles a second, to reach us from certain stars. This causes one to believe, with the ancients, that the microcosm is as great as the macrocosm.

Some little can be gained of what is meant by thinking of a grain of substance so thoroughly divided as to be dissolved equally in a body of water the bulk of our earth. Almost any one, seriously saying to another who did not know the truth of the matter, that they believe in the curative action of a few drops of the wonderful fluid prepared in this way, or giving it to the sick, would be looked upon with pity and derision as a lunatic. The truth of this principle, concerning potencies, so persistently and constantly advocated by the founder of Homeopathy, bears directly on everything he said and did, especially during his later years. It should be added that probably only a small minority of his professed followers practice according to these views. The larger number, while they honor him for his services to rational, exact, and scientific therapeutics, do not hold them. To the mind just awakening, the facts and figures of astronomy are just as ridiculous and beyond belief as the teachings of this wondrous man to the materialist in medicine. A competent investigation will alone convince him. The most taking argument against the new system, to an inquirer, is, that it is like putting a drop of medicine into the ocean—that no medicine is given. Any one who has laboriously and frequently worked out this principle of making potencies knows that it is not dilution merely. A great number of shakings, using only small quantities of a solvent at a time, and going on step by step, upwards in the scale, is something entirely and altogether different from it. A force is evolved gradually, by separation of the atoms and retained by the menstruum. This, like most facts, cannot be known beforehand; it must be put to the severe test of provings on the healthy and cures of the sick—not once, but many times, so that the investigator may determine the facts for himself. Not only will medicines prepared in this manner produce constant, but will, in suitable cases, cure similar symptoms found in the sick. Ridicule, abuse, previous notions, or prejudice are not arguments, and will not answer, and no physician can properly believe in the cardinal principles of the new system unless he himself has prepared his own medicines, and made his own provings therefrom.

His lifetime would not suffice to do but a small part of all that has been done, but he must have done enough of this kind of work over and over again, so as to be sure that his future is not to be based on false principles. The testimony of the founder should be his warrant for investigation.

Some will and do say, "Of what use is all this; cannot the crude or massive doses be given according to the principle of the similars?" Yes! but better work, more thorough and quicker cures without accompanying medicinal disturbances or disease, will be made by Hahnemann's methods. In using potencies one must have an interior or intimate personal knowledge of them, and that not altogether from books. Nothing is accomplished by a faulty selection, and one lacking in this can probably do better with sensible doses of the crude drug because more symptoms are in apparent harmony with the disease.

The trend of medical practice throughout the world is toward the employment of single remedies in small appreciable doses. It is thought that the time is coming, as it did with Hahnemann himself, when the potentized medicine will come into general use, as a result of diluting the dose. The practice is now generally ridiculed, but, if correct, it will eventually win. A serious objection, with many, to their use, is the fact that you cannot tell whether or not they have been spoiled by contact with other similar preparations, except by proving. They require excessive care in handling, but richly repay one in their astounding curative results.—*Hospital Tidings*, March, 1897.

